# Enhancement of Angiogenesis by Ultrasound-Targeted Microbubble Destruction Combined with Nuclear Localization Signaling Peptides in Canine Myocardial Infarction

**DOI:** 10.1155/2017/9390565

**Published:** 2017-11-12

**Authors:** Jingjing Cui, Qing Deng, Qing Zhou, Sheng Cao, Nan Jiang, Yijia Wang, Jinling Chen, Bo Hu, Tuantuan Tan

**Affiliations:** ^1^Department of Ultrasound Imaging, Renmin Hospital of Wuhan University, Wuhan 430000, China; ^2^Department of Ultrasound Imaging, Affiliated Hospital of Jining Medical University, Jining 272000, China

## Abstract

**Objective:**

This study aimed to develop a gene delivery system using ultrasound-targeted microbubbles destruction (UTMD) combined with nuclear localization signal (NLS) and investigate its efficacy and safety for therapeutic angiogenesis in canine myocardial infarction (MI) model.

**Methods:**

Fifty MI dogs were randomly divided into 5 groups and transfected with Ang-1 gene plasmid: (i) group A: only injection of microbubbles and Ang-1 plasmid; (ii) group B: only UTMD mediated gene transfection; (iii) group C: UTMD combined with classical NLS mediated gene transfection; (iv) group D: UTMD combined with mutational NLS mediated transfection; and (v) group E: UTMD combined with classical NLS in the presence of a nucleus transport blocker. The mRNA and protein expression of Ang-1 gene, microvessel density (MVD) cardiac troponin I (cTnI), and cardiac function were determined after transfection.

**Results:**

The expression of mRNA and protein of Ang-1 gene in group C was significantly higher than that of the other groups (all* P* < 0.01). The MVD of group C was 10.2-fold of group A and 8.1-fold of group E (*P* < 0.01). The cardiac function in group C was significant improvement without cTnI rising.

**Conclusions:**

The gene delivery system composed of UTMD and NLS is efficient and safe.

## 1. Introduction

Since the first gene therapy trial was launched at the clinical center of the National Institute for Health (NIH) using a retrovirus for treating severe combined immunodeficiency disease [1], gene therapy has been considered a prospective tool for difficult-to-treat diseases by using the clinical techniques that are currently available [2]. Ultrasound-targeted microbubble destruction (UTMD), which is a new nonviral vector system capable of increasing transmembrane transport through sonoporation, has been shown to provide targeted delivery of reporter genes, therapeutic genes, and antisense oligonucleotides to tissues and organs in a safe manner [3, 4]. However, the potential applications for this method are still a challenge due to the poor transfection efficiency of the exogenously transferred genes* in vivo* [5, 6].

Poor transfer of DNA fragments from the cytoplasm to the nucleus is one of the major reasons for the low exogenous gene transfection efficiency [7, 8]. Labat-Moleur et al. [9] showed that only 1 plasmid DNA fragment per 100 fragments could enter the nucleus from the cytoplasm in the gene transfer with lipopolyamines. It has been acknowledged that the outcomes of gene therapy are highly associated with the delivery of transferred DNA to the nucleus. However, previous studies of therapeutic gene delivery using UTMD have paid more attention to increasing the penetration of the cell membrane via physical bioeffects [10, 11], and few studies have been carried out to target gene breakthrough in the nuclear barrier during UTMD transfection in an effective manner.

It is well known that exogenous DNA can be transferred into the nucleus via active transport facilitated by the nuclear localization signal (NLS) [12, 13]. Active transport is an energy-consuming process. For the mechanism of UTMD, microbubbles may serve as cavitation nuclei to accomplish sonoporation during ultrasound irradiation which creates transient and reversible pores on the cell membrane. A large amount of energy was released during the process which could provide the energy needed for nuclear transport of NLS theoretically [14]. Therefore, it would be possible to develop a nucleophilic gene transfection system based on UTMD and NLS.

Angiogenin-1 (Ang-1) gene encodes the angiopoietin 1, which is involved in the specific chemiotaxis of the endothelial cells* in vivo* and thereby mediates the migration of endothelial cells to injury sites [15]. Further, the gene contributes to elevating the binding affinity of both endothelial cells and the basement membrane, which consequently induce de novo capillary recruitment and decrease the permeability of the capillaries. Based these factors, this study aimed to develop a novel gene transfection system composed of UTMD and NLS and to investigate the therapeutic potential of this system for treating ischemic heart disease by transfecting the Ang-1 gene into a canine myocardial infarction model.

## 2. Materials and Methods

### 2.1. Animal Model of Myocardial Infarction

Male adult hybrid dogs (weighing 17.3 ± 3.2 kg) were supplied by the Animal Experimental Center of Wuhan University (Approval number SYXK2004-0027, Wuhan, China). The study protocols were approved by the Ethical Committee of Renmin Hospital of Wuhan University. The experiments were performed according to the Guide to the Care and Use of Experimental Animals of US National Institutes of Health.

The dog myocardial infarction (MI) model was induced using interventional embolization. Briefly, the dogs in a fasting state were anesthetized with pentobarbital sodium (30 mg/kg) followed by a tracheal cannula. After femoral artery puncture, a 6 F sheathing canal was inserted, followed by an infusion of heparin (1000 U). Then, a 5 F catheter was inserted into the ostium of the left coronary artery, followed by confirmation using DSA (Philips FD20, Andover, MA, USA). Subsequently, the microguide wire and catheter were inserted through the 5 F catheter until the orifice of the second diagonal branch of left anterior descending artery (LAD) was reached. Then, gelfoam granules (250 *μ*m, Alicon, Hangzhou, China) were infused into the distal part of the second diagonal branch of LAD to block the blood circulation. MI was confirmed by an elevation of ST segments in the ECG.

### 2.2. Experimental Group

The animals with successful MI induction were randomly divided into 5 groups with 10 in each group.

*Group A*. It is control, given an intravenous injection of 1 ml of SonoVue microbubbles (Bracco, Geneva, Switzerland), 1 ml of Ang-1-EGFP plasmid (0.5 mg/ml; Gene Chem, Shanghai, China), and 1 ml of saline. 

*Group B*. It is ultrasound-mediated plasmid DNA transfection group, which was treated with a 1 ml suspension of the SonoVue microbubbles, 1 ml of Ang-1-EGFP plasmid, and 1 ml of saline. 

*Group C*. It is ultrasound combined with classic nuclear localization signal- (cNLS-) mediated plasmid DNA transfection, which was subjected to a suspension of 1 ml of SonoVue microbubbles, 1 ml ofAng-1-EGFP plasmid, and 1 ml of cNLS (PKKKRKV; 0.67 mg/ml; Shanghai Science Peptide BioTech, Shanghai, China). 

*Group D*. It is ultrasound combined with mutational nuclear localization signal- (mNLS-) mediated plasmid DNA transfection, which was treated with a mixture of 1 ml of SonoVue microbubbles, 1 ml of Ang-1-EGFP plasmid, and 1 ml of mNLS (PKTKRKV; 0.67 mg/ml; Shanghai Science Peptide BioTech, Shanghai, China) for transfection. 

*Group E*. It is ultrasound combined with cNLS-mediated plasmid DNA transfection while adding wheat germ agglutinin (WGA, which is a nucleus transport blocker, Sigma-Aldrich, CA, USA). The transfection suspension consisted of 1 ml of SonoVue microbubbles, 1 ml of Ang-1-EGFP plasmid, 1 ml of cNLS, and 0.3 ml of WGA (0.1 mg/ml). 

In this study, a SonoVue microbubble suspension was prepared according to the manufacturer's instruction manual. The microbubbles were filled with sulfur hexafluoride gas and encapsulated by a thin and flexible phospholipid monolayer. The average diameter of the microbubbles was approximately 2.5 *μ*m, and the concentration was 2 × 10^8^–5 × 10^8^/ml. The Ang-1-EGFP plasmid was constructed by GeneChem Technology Co., Ltd. (Shanghai, China). In this plasmid, the coding region of Ang-1 was cloned in the vector GV230, under the control of the human cytomegalovirus promoter/enhancer followed by the enhanced green fluorescent protein (EGFP), for visualization of protein expression. The constructed plasmid was transformed into* E. coli* strain DH5a for amplification and purified using the Wizard™ Maxiprep DNA Purification System (Promega Corporation, Madison, WI, USA). The NLS peptide was purified by high-performance liquid chromatography, and the optimal molar ratio of NLS and Ang-1-EGFP plasmid was 10^4^ : 1, according to our previous experiments [16]. The suspension of NLS and Ang-1-EGFP plasmid was incubated at room temperature for 30 minutes and added to 1 ml of SonoVue microbubble suspension. The mixture was incubated for 2 hrs at room temperature, and the upper layer suspension of microbubbles attached to plasmid and NLS was used for intravenous injection.

### 2.3. Gene Transfection

Gene transfection was carried out 24 hrs after MI via intravenous injection of 3 ml of various suspensions according to the above group setting. UTMD was performed using a Vivid Q ultrasound diagnostic platform (GE Healthcare, WI, USA) equipped with a M5S transducer (1.7–3.3 MHz). The parasternal short axis view at the level of papillary muscles was selected for ultrasound irradiation. The ultrasound exposure started right after microbubble injection, and it terminated when the intramyocardium microbubbles completely vanished. The mechanical index was set at 1.3, and the gain was modulated to the maximal level during ultrasound irradiation.

Three days after transfection, three dogs from each group were sacrificed to collect the myocardium in the peripheral infarction area for the freezing section, real-time PCR, and Western Blot analysis. Twenty-eight days after transfection, seven dogs from each group were sacrificed to obtain the myocardium for the evaluation of the area of MI, Masson staining, and immunofluorescent analyses.

### 2.4. Freezing Section Analysis

Frozen sections were prepared based on the myocardium stored at −80°C to detect the expression of Ang-1-EGFP plasmid. The sections were then observed under a BX51 fluorescence microscope (Olympus, Tokyo, Japan) to evaluate the expression of EGFP-labeled Ang-1 plasmid. The excitation wavelength was set at 488 nm. The tests were performed in triplicate.

### 2.5. Real-Time PCR

Total RNA was extracted from the myocardium (100 mg) using Trizol (Invitrogen, CA, USA) according to the manufacturer's instructions. The cDNA synthesis was carried out with approximately 2 *μ*g of RNA using the PrimeScript™RT reagent Kit with gDNA Eraser (Takara, Dalian, China). Real-time PCR was conducted using SYBR Green with the primers (5′-GAAGGAAACCGAGCCTATTCAC-3′ and 5′-CCACAAGCATCAAACCACCA-3′) synthesized by Invitrogen (Shanghai, China) according to the previous study [16]. The mRNA level was normalized by GAPDH. PCR reactions were performed in a total volume of 10 *μ*l, containing 5 *μ*l of 2x SYBR Premix, 0.2 *μ*l of each specific primer to a final concentration of 200 *μ*l, and 1 *μ*l of cDNA template. The PCR conditions consisted of denaturation at 95°C for 1 minute, followed by 40 cycles of denaturation at 95°C for 15 s, annealing at 58°C for 20 s and extension at 72°C for 45 s. The amplification results for real-time PCR were calculated as 2(−ΔΔCt) [17].

### 2.6. Western Blot Analysis

The myocardium (100 mg) was homogenized in RIPA lysis buffer containing protease and phosphatase inhibitors. Proteins were separated by electrophoresis on an SDS-PAGE gel and transferred to a PVDF membrane. The membrane was blocked in 5% nonfat milk and incubated with anti-Ang-1 antibody (1 : 1000; ab133425; Abcam, Cambridge, MA, USA) overnight at 4°C; then it was incubated with the HRP-conjugated IgG (1 : 2000; ab97057; Abcam, Cambridge, MA, USA) for 1 h at room temperature. The protein band was evaluated using the ECL chemiluminescence system (KeyuShenlan Scientific Co., Ltd., Beijing, China). The housekeeping protein (GAPDH) was used as the internal standard for the Ang-1 calculation. The ratio of protein/GAPDH was used to quantify the Ang-1 protein levels in each group.

### 2.7. Microvascular Density

The density of microvessels was determined using immunohistochemistry for alpha smooth muscle actin (*α*-SMA). To estimate microvascular density, capillaries and arterioles were counted in five fields per slide using fluorescence microscopy (200x; BX52, Olympus, Japan), and the average value was taken.

### 2.8. Cardiac Function Evaluation

Two-dimensional echocardiography was performed before transfection (baseline) and 28 days after transfection. The echo instrument was GE Vivid Q, as mentioned earlier. The left ventricular end-diastolic dimension (LVEDD) and left ventricular end-systolic dimension (LVESD) were measured, and the left ventricular end-diastolic volume (LVEDV) and left ventricular end-systolic volume (LVESV) as well as the left ventricular ejection fraction (LVEF) were obtained by biplane Simpson's method.

### 2.9. Determination of Myocardial Fibrosis Degree and Infarction Size

Masson staining was performed to evaluate myocardial fibrosis. The normal myocardial fiber is shown in red, and the collagen fiber was shown in blue to distinguish normal myocardium from fibrosis tissue.

Regarding the evaluation of the infarct area, the excised heart was washed with normal saline, followed by removal of the atrium and auricle of the heart as well as the right ventricle. From the apex to the basal level, the left ventricle was transversely cut into five sections with an average thickness of 0.8–1.0 cm. The myocardial tissue at the level of the papillary muscle for each group was photographed using a digital camera; then the images were analyzed using Image-ProPlus 6.0 software (Media Cybernetics, MD, USA). Areas in a white color were defined as the myocardial infarct zone. The ratio of the infarct area to the total area of the left ventricular at the papillary muscle level was calculated to assess the size of infarction after treatment.

### 2.10. Serum cTnI Determination

To determine the safety of the gene delivery system composed of UTMD and NLS, the level of myocardial enzyme cardiac Troponin I (cTnI) before and after transfection of each group was detected. Blood samples were collected from the ulnar vein at the time points before MI and 4 hrs, 24 hrs after MI (pre-MI, MI-4 h, MI-24 h), and 1 day and 6 days after transfection (Trans-D1, Trans-D6), respectively. The serum cTnI was detected with the commercial kit using the Beckman Coulter Immunochemiluminescent system.

## 3. Statistical Analysis

The SPSS 20.0 and GraphPad 6.01 software packages were used for the data analysis. Measurement data were presented as the mean ± standard deviation. The K-S test was used to determine the normal distribution of the data. ANOVA was used to compare multiple groups, and the LSD method was used for comparisons between two groups. *P* < 0.05 was considered statistically significant.

## 4. Results

### 4.1. Mortality of Animal Model

**Figure 1:**
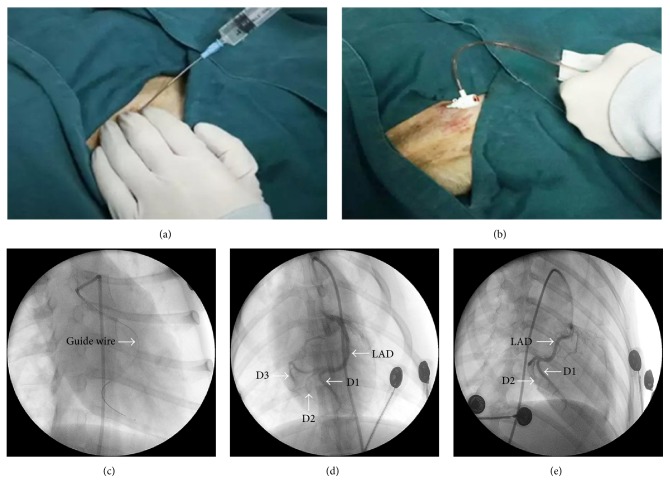
Schematic diagram of the myocardial infarction (MI) model using coronary artery embolism. (a) The puncture was performed at the obvious pulse location on the right femoral artery. (b) The artery sheath was inserted after puncture. (c) After the angiographic catheter reached the opening of the left coronary artery, the microguide wire and catheter could access the left anterior descending coronary artery. (d) The left coronary artery branches were clearly displayed before the MI modeling by coronary angiography. D1 = the first diagonal branch; D2 = the second diagonal branch; and D3 = the third diagonal branch. (e) After embolism of the coronary artery, the second diagonal branch distal of the left anterior descending artery was blocked. The second coronary angiography confirmed ischemia in the embolism area.

Fifty-eight dogs received MI induction, among which two died from an anesthetic accident, three died from ventricular fibrillation, and three died died from operation. Finally, fifty dogs were confirmed with MI featured by elevation in the ST segment and blood circulation blockage on coronary angiography (Figure 1).

### 4.2. Plasmid Expression

**Figure 2:**
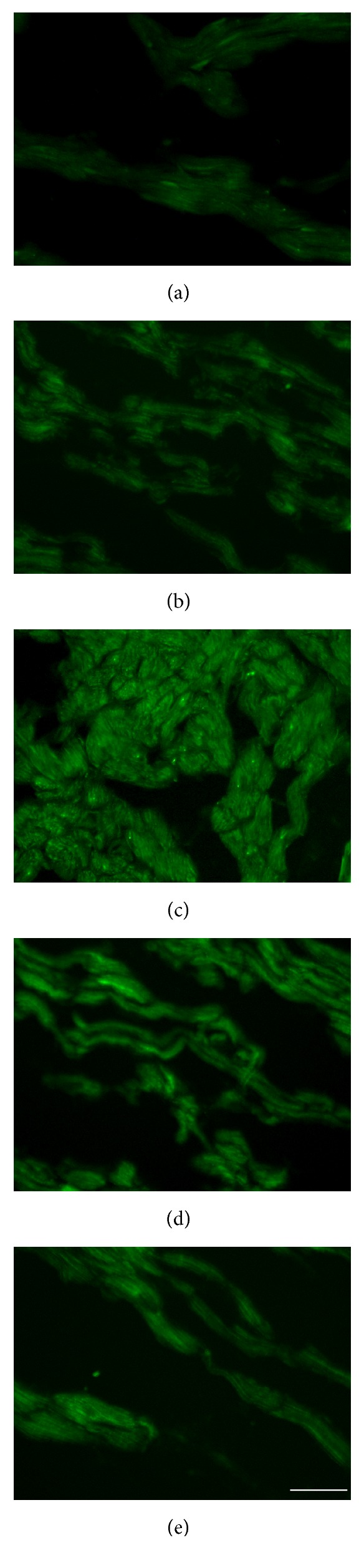
Representative micrographs for the expression of enhanced green fluorescent (EGFP) protein. EGFP in the border regions of MI was detected by confocal laser scanning microscopy on day 28 after transfection. The images were observed under a magnification of 400x. Scale bar = 50 *μ*m. (a), (b), (c), (d), and (e) represented groups A, B, C, D and E, respectively.

Frozen section analysis showed EGFP was expressed in all five groups. The expression of EGFP was gradually increased from group A to group C and decreased from group C to group E. The expression of EGFP peaked in group C (Figure 2).

**Figure 3:**
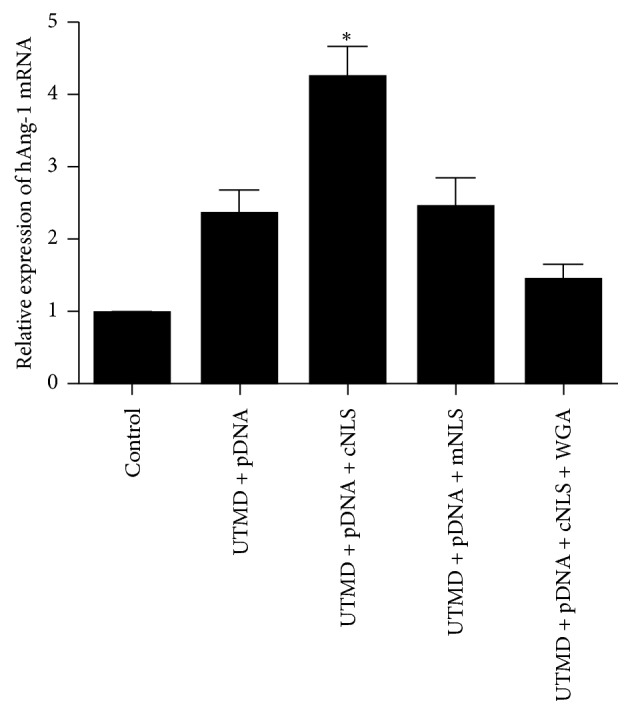
Real-time PCR detects Ang-1 mRNA expression in the MI regions of canines 3 days after transfection. The Ang-1 mRNA was normalized by GAPDH. The Ang-1 mRNA was higher in group C compared to the other groups, ^*∗*^*P* < 0.05 compared to the other groups.

**Figure 4:**
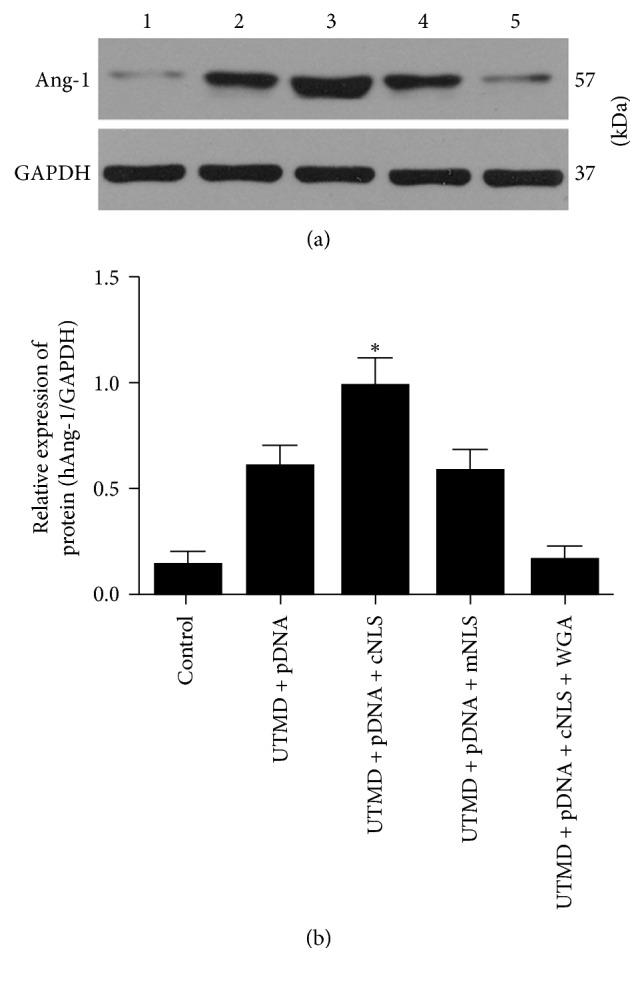
Western Blot analysis of Ang-1 protein expression in the MI border regions of canines. (a) Myocardial Ang-1 and GAPDH protein levels quantified using a Western Blot 3 days after UTMD-mediated Ang-1 transfection. Lanes 1–5 represented groups A, B, C, D, and E, respectively. (b) Quantification of the Ang-1 protein relative expression levels was performed in the MI border regions of canines in all groups. The Ang-1 was normalized by GAPDH. The Ang-1 protein expression was the highest in group C; ^*∗*^*P* < 0.05 compared to the other four groups.

Real-time PCR and Western Blot all showed that the expression of Ang-1 gene and protein were consistent with the expression of green fluorescent protein. For groups A, B, C, D, and E, the relative expressions of Ang-1 mRNA were (1.0 ± 0.01), (2.36 ± 0.33), (4.25 ± 0.43), (2.44 ± 0.42), and (1.43 ± 0.23), respectively, and the relative expressions of Ang-1 protein were (0.15 ± 0.06), (0.61 ± 0.10), (0.99 ± 0.13), (0.59 ± 0.10), and (0.16 ± 0.07), respectively. The relative expression of both Ang-1 mRNA and protein in group C was higher than in the other groups, with statistical significance (*P* < 0.05, all, Figure 3). The relative expression of Ang-1 protein in group C was 1.6-fold higher than group B, which indicated that UTMD combined with NLS could effectively promote gene expression than UTMD alone (Figure 4). The closely equivalent results of groups B and D showed that the mutated NLS did not promote gene expression due to its loss of capacity for nuclear transport. The comparison of groups C and E indicated that the addition of WGA blocked nuclear transport and significantly decreased the expression of Ang-1 protein.

### 4.3. Microvascular Density

**Figure 5:**
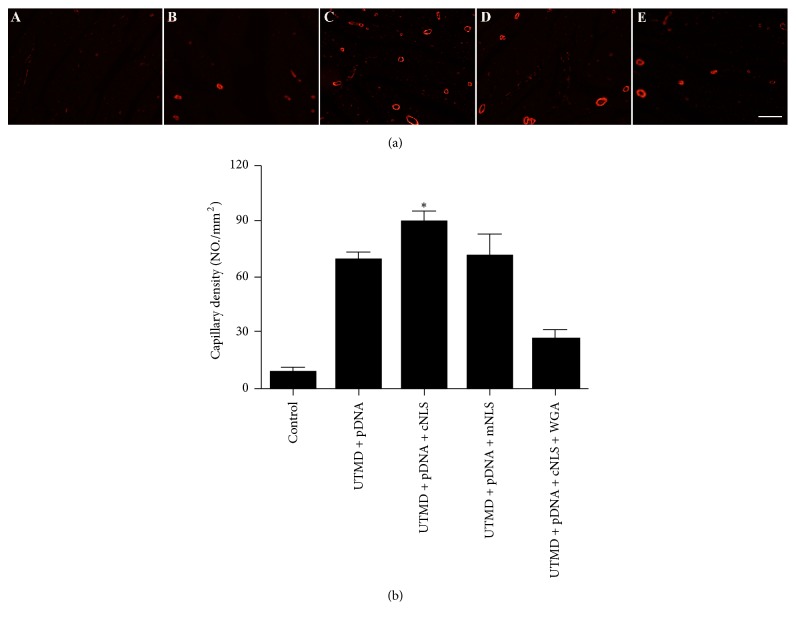
Angiogenesis after UTMD-mediated Ang-1 transfection in the MI border regions on day 28. (a) Representative images of capillary density from all groups on day 28 after transfection. The images were observed under a magnification of 200x. Scale bar = 20 *μ*m. (b) The quantification of capillary density in group C was significantly higher compared to the other groups (^*∗*^*P* < 0.01).

The newly formed capillary vessels were stained a red color by immunohistochemistry for *α*-SMA. The density of the microvessels of group A, B, C, D, and E was (8.8 ± 2.8/mm^2^), (69.0 ± 3.7/mm^2^), (89.3 ± 6.1/mm^2^), (71.3 ± 11.6/mm^2^), and (11.0 ± 4.3/mm^2^), respectively. The microvascular density in group C was statistically higher than that in the other four groups (Figure 5).

### 4.4. Cardiac Function Improvement

No significant differences were noticed in the dimension or volume of the left ventricle or in LVEF among all five groups (*P* > 0.05) before transfection. The LVEF of groups A, B, C, D, and E at day 28 after gene transfection was (29.7 ± 5.8%), (41.6 ± 2.6%), (46.9 ± 2.7%), (40.4 ± 2.6%), and (33.6 ± 2.0%), respectively. Compared to the LV systolic function at baseline, the LVEF of groups A and E was further decreased, whereas that of groups B and D was slightly increased. The LVEF of group C showed a significant improvement compared to the other groups after transfection (*P* < 0.01, Table 1).

### 4.5. Myocardial Fibrosis and Infarct Size Assessment

**Figure 6:**
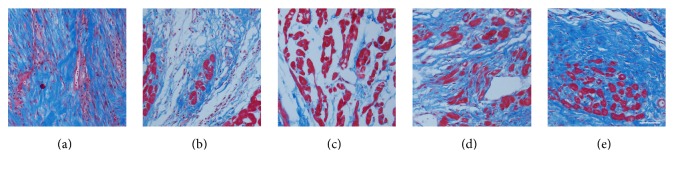
Masson trichrome staining of the infarcted zone on day 28 after Ang-1 gene transfection. Representative photomicrographs show the fibrosis of infarcted myocardium (myocardium in red, fibrosis in blue, magnification 100x, scale bar = 100 *μ*m). Compared with other groups, fibrosis of the infarct zone was obviously suppressed in group C, which was featured by less collagen fiber and formation of more normal myocardium. (a), (b), (c), (d), and (e) represented groups A, B, C, D, and E, respectively.

Masson staining revealed that the degree of myocardial fibrosis in the five groups decreased from A to C, and groups D and E were more severe than group C. The myocardial fibrosis in group C was the least serious among the five groups (Figure 6).

**Figure 7:**
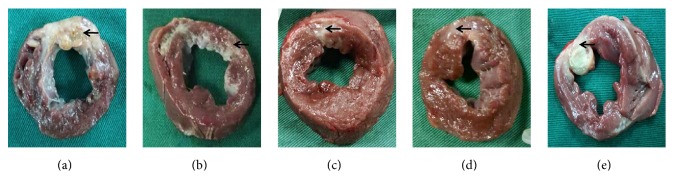
Pathomorphology of infarcted myocardium after Ang-1 gene transfection. Pathological specimens of the left ventricular short axis at the papillary muscle level from all groups on day 28 after Ang-1 transfection in canines. The tissue in white (marked by black arrow) indicated infarcted myocardial, and the tissue in red represented the noninfarcted myocardium. A wide range of tissue in white indicated more MI area in (a), whereas a higher percentage of tissue in red indicated noninfarcted myocardium in (c). (a), (b), (c), (d), and (e) represented groups A, B, C, D, and E, respectively.

Similarly, the MI area in group C was the lowest among the five groups (Figure 7). The infarct areas of groups A, B, C, D, and E accounted for approximately 1/2, 1/4, 1/5, 1/4, and 1/3 of the left ventricular area, respectively. These results indicated that UTMD combined with NLS-mediated Ang-1 gene delivery contributed to the attenuation of the myocardial fibrosis and MI area.

### 4.6. Determination of cTnI

The change of the level of the serum cTnI showed the same trend in all five groups. The cTnI significantly increased at 24 hrs after MI and gradually decreased. There was no significant difference in cTnI before and after gene transfection (*P* > 0.05), which indicated that this gene transfection system did not cause extra damage to cardiomyocytes compared to simple UTMD gene delivery (Table 2).

## 5. Discussion

Refractory ischemic heart disease is a severe disease with high mortality and morbidity and affects public health and living quality worldwide. Currently, UTMD-mediated gene delivery for neovessel formation is considered a promising nonviral system for treating myocardial ischemia [18, 19]. In this study, we investigated the efficiency of UTMD combined with NLS for treating myocardial ischemia. The novelty of the study is that the UTMD-NLS gene delivery system allows gene transport with dual-targeting in terms of membrane and nuclear barriers and can achieve a better improvement of the angiogenesis and cardiac function than can UTMD transfection alone.

Three major factors have been reported to be responsible for the poor transfection efficiency of exogenous genes, including the cell membrane barrier, cytoplasmic degradation, and the nuclear membrane barrier [20]. To date, few studies have confirmed that ultrasound irradiation can release DNA into the cytoplasm and pass through the nuclear membrane, regardless of microbubble destruction, as long as the ultrasonic frequency is high enough and the total radiation energy is sufficient. However, this process has a long duration (30 min), which requires ultrasound irradiation, and the cell survival rate is lower than 80% [21]. In this study, a functional group (termed as NLS) was experimentally utilized in combination with the UTMD technique with the aim of improving the gene transferring efficiency instead of long-term ultrasound exposure.

NLS, which is a functional peptide derived from eukaryotic nucleoprotein and viral protein, could effectively mediate the intranuclear transport of molecules [22]. PKKKRKV, which is the minimum NLS of SV40T antigen, has been commonly used in the construction of nonviral genetic vector because it shows high transfection efficiency and low immunogenicity [23, 24]. For example, Park et al. transfected the human lipocytes using PEI-NLS-GFP labeled plasmid, which showed higher gene transfer potency than the non-NLS group, even on day 7 [25]. At the same time, they reported that NLS contributed to the stability of exogenous DNA fragments by preventing the degradation of transferred genes in cytoplasm [26]. Jeon et al. transfected the human skin fibroblasts using poly (lactide-co-glycolide) (PLGA) combined with NLS and plasmid DNA, which indicated that NLS conjugation enhanced the gene transfection efficiency by 1.2–~3.2-fold over 13 days* in vitro*, and the pDNA was released from PLGA nanospheres over 9 days [27]. In this study, a gene delivery system was constructed based on the biological function of UTMD and NLS. Our results showed that the transfection efficiency of the Ang-1 gene was approximately 1.6-fold compared to the UTMD system alone in the ischemic myocardium of dogs. Additionally, the number of neovessels formed in the MI region increased, along with reduced myocardial scarring and fibrosis. Moreover, the cardiac function was significantly improved. Based on this outcome, we considered that the system was nucleophilic and could target both the cell membrane and nuclear membrane. To further testify the roles of NLS in the delivery system, a mutational NLS was used. As shown previously, an accurate NLS sequence and structure are the premise for the transport of a receptor protein and nuclear pore complex. However, in the presence of mutation in ^126^PKKKRKV^132^, the linkage and amino acid sequences may change, which results in transport loss into the nucleus because the mutational NLS is not recognized by the receptor [28, 29]. In addition, to clarify the nuclear transport of cNLS, WGA was used to block the nuclear pore, which then obstructed the binding of the NLS and the receptors. Although WGA did not affect the binding of plasmid DNA and NLS, the DNA could not be transported into the nucleus [30, 31]. Our results showed that mNLS or WGA could decrease the Ang-1 transfection efficiency and protein expression. The expression of the Ang-1 protein in group C was approximately 1.7-fold that of group D and 6.2-fold that of group E. This outcome indicated that dysfunction and/or blocking of NLS would significantly impair the delivery of therapeutic genes.

Concerning the gene delivery impact, the combination mode of NLS and plasmid DNA needs to be considered. NLS contained basic amino acids with positive charges under neutral conditions and could couple DNA with negative charges through electroadsorption. According to a previous study, the binding of NLS and DNA via electrostatic interaction could facilitate the entry of nonnuclear protein into the nucleus [32]. The disadvantage of this coupling mode was the possibility of the dissociation of NLS and DNA, which weakened the DNA in the nucleus. To strengthen the combination, the covalent binding mode was used in some studies, but special attention should be paid to prevent NLS from combining with functional gene expression components; otherwise, the gene cannot be well-expressed [33]. Based on this approach, we chose electrostatic binding in this study, which was easy to perform and could maintain the integrity of the NLS and DNA fragment [34].

Regarding potential applications, it is necessary to determine the safety while enhancing the efficiency of gene expression. The difference of cTnI was not significant on day 1 or day 6 after transfection among all groups, which suggested that NLS did not cause extra cardiomyocytes injury under UTMD. The cell injury during the gene delivery might be associated with the increased cell membrane permeability caused by cavitation effects of UTMD, whereas, in this study, it was depending on the function of NLS rather than on microbubbles or long-duration ultrasound exposure to help DNA enter the nucleus; thus the negative bio-effects on the cell should not be more severe than that observed when using UTMD alone.

There are some limitations to this study. Although NLS has been extensively utilized in molecular research, its roles in gene delivery into the nucleus are still not well defined. Therefore, we could only focus on the therapeutic impact without illustrating the potential mechanism. In addition, the transfection parameters still need to be explored further because we only optimized the molar ratio of NLS to plasmid (10^4^ : 1) based on a previous description [35] and our previous data. More experiments are needed to determine the collocation among transfection parameters, such as ultrasound exposure (e.g., irradiation energy, density, and duration) and NLS concentration.

## 6. Conclusions

In conclusion, the nucleophilic delivery system constructed from the combination of UTMD and NLS contributed to the transfection efficiency by promoting a therapeutic gene in the nucleus and effectively increasing the angiogenesis in a canine myocardial infarction model.

## Figures and Tables

**Table 1:** Cardiac function in each group 28 days after treatment $\left(\overset{-}{x}\pm s\right)$.

Groups	LVEDD (mm)		LVESD (mm)		LVEDV (ml)		LVESV (ml)		LVEF (%)	
	Baseline	28 d	Baseline	28 d	Baseline	28 d	Baseline	28 d	Baseline	28 d
Group A	27.1 ± 3.1^ce^	29.3 ± 2.9^c^	22.4 ± 2.5	25.3 ± 1.7	29.6 ± 9.4	33.2 ± 8.0^c^	18.4 ± 5.7	23.1 ± 4.1	38.0 ± 2.4	29.7 ± 5.8^bcd^
Group B	28.9 ± 1.2	31.0 ± 1.6^c^	24.0 ± 1.2	24.7 ± 1.5^e^	32.0 ± 3.2	37.6 ± 4.6^c^	20.0 ± 2.3	22.0 ± 3.5^e^	37.6 ± 2.6	41.6 ± 2.6^ace^
Group C	30.9 ± 1.6^ad^	33.4 ± 1.1^abde^	25.3 ± 1.7	26.0 ± 0.8^d^	37.3 ± 4.5	45.0 ± 3.8^abde^	23.1 ± 3.9	23.6 ± 2.3^d^	37.3 ± 3.1	46.9 ± 2.7^bde^
Group D	28.3 ± 2.3^c^	29.3 ± 2.4^c^	23.6 ± 1.7	23.7 ± 2.0^ce^	30.7 ± 6.3	33.4 ± 6.7^c^	19.3 ± 3.8	19.9 ± 4.0^ce^	36.8 ± 3.6	40.4 ± 2.6^ace^
Group E	29.9 ± 2.3^a^	31.0 ± 1.4^c^	24.9 ± 1.9	26.6 ± 1.0^bd^	34.9 ± 6.2	37.8 ± 4.0^c^	22.0 ± 4.1	26.1 ± 2.5^bd^	36.9 ± 3.1	33.6 ± 2.0^bcd^
*F*	2.959	5.040	2.600	4.065	1.66	4.932	1.580	3.292	0.107	33.173
*P*	0.036	0.003	0.056	0.009	0.162	0.004	0.205	0.024	0.979	0.000

LVEF = left ventricular eject fraction, LVEDD = left ventricular end-diastolic dimension, LVESD = left ventricular end-systolic dimension, LVEDV = left ventricular end-diastolic volume, and ESV = left ventricular end-systolic volume. Values are means ± SD. ^a^*P* < 0.05, compared with group A; ^b^*P* < 0.05, compared with group B; ^c^*P* < 0.05, compared with group C; ^d^*P* < 0.05, compared with group D; and ^e^*P* < 0.05, compared with group E.

**Table 2:** Serum cTnI at different time points in each group $\left(\overset{-}{x}\pm s\right)$.

Groups	pre-MI	MI-4 h	MI-24 h	Trans-D1	Trans-D6
Group A	49.6 ± 4.4	72.6 ± 6.9	147.4 ± 6.8	68.6 ± 4.8	53.6 ± 3.8
Group B	53.0 ± 5.4	70.6 ± 7.5	152.0 ± 5.6	67.6 ± 5.5	54.4 ± 4.6
Group C	56.6 ± 5.9	71.8 ± 7.2	153.8 ± 3.4	69.6 ± 4.0	56.0 ± 5.4
Group D	56.4 ± 5.9	72.8 ± 9.6	153.6 ± 7.3	67.6 ± 3.4	55.6 ± 7.4
Group E	56.6 ± 5.3	71.8 ± 9.09	158.8 ± 7.2	76.0 ± 8.6	58.4 ± 4.3
*F*	1.653	0.057	2.151	2.003	0.608
*P*	0.200	0.994	0.112	0.133	0.662

Pre-MI = before myocardial infarction, MI-4 h = 4 hours after myocardial infarction, MI-12 h = 12 hours after myocardial infarction, Trans-D1 = 1 day after transfection, and Trans-D6 = 6 days after transfection. Values are means ± SD.
